# Factors Influencing Care Pathways for Breast and Prostate Cancer in a Hospital Setting

**DOI:** 10.3390/ijerph18157913

**Published:** 2021-07-26

**Authors:** Ornela Bardhi, Begonya Garcia-Zapirain, Roberto Nuño-Solinis

**Affiliations:** 1 eVida Laboratory, University of Deusto, Avenida Universidades 24, 48007 Bilbao, Spain; mbgarciazapi@deusto.es; 2 UCD Beacon Hospital Academy, Beacon Hospital, Sandyford, D18 AK68 Dublin, Ireland; 3 Deusto Business School Health, University of Deusto, Avenida Universidades 24, 48007 Bilbao, Spain; roberto.nuno@deusto.es

**Keywords:** breast cancer, prostate cancer, care pathways, treatment lines, lifestyle data

## Abstract

Breast cancer (BCa) and prostate cancer (PCa) are the most prevalent types of cancers. We aimed to understand and analyze the care pathways for BCa and PCa patients followed at a hospital setting by analyzing their different treatment lines. We evaluated the association between different treatment lines and the lifestyle and demographic characteristics of these patients. Two datasets were created using the electronic health records (EHRs) and information collected through semi-structured one-on-one interviews. Statistical analysis was performed to examine which variable had an impact on the treatment each patient followed. In total, 83 patients participated in the study that ran between January and November 2018 in Beacon Hospital. Results show that chemotherapy cycles indicate if a patient would have other treatments, i.e., patients who have targeted therapy (25/46) have more chemotherapy cycles (95% CI 4.66–9.52, *p* = 0.012), the same is observed with endocrine therapy (95% CI 4.77–13.59, *p* = 0.044). Patients who had bisphosphonate (11/46), an indication of bone metastasis, had more chemotherapy cycles (95% CI 5.19–6.60, *p* = 0.012). PCa patients with tall height (95% CI 176.70–183.85, *p* = 0.005), heavier (95% CI 85.80–99.57, *p* < 0.001), and a BMI above 25 (95% CI 1.85–2.62, *p* = 0.017) had chemotherapy compared to patients who were shorter, lighter and with BMI less than 25. Initial prostate-specific antigen level (PSA level) indicated if a patient would be treated with bisphosphonate or not (95% CI 45.51–96.14, *p* = 0.002). Lifestyle variables such as diet (95% CI 1.46–1.85, *p* = 0.016), and exercise (95% CI 1.20–1.96, *p* = 0.029) indicated that healthier and active BCa patients had undergone surgeries. Our findings show that chemotherapy cycles and lifestyle for BCa, and tallness and weight for PCa may indicate the rest of treatment plan for these patients. Understanding factors that influence care pathways allow a more person-centered care approach and the redesign of care processes.

## 1. Introduction

According to the World Health Organization, it is estimated that by 2040 the number of incident cases will be 28.9 million for all cancer, an increase of approximately 11.3 million new cases [1]. The number of incident cases for breast cancer (BCa) (female) is estimated to increase by 764,052 new cases (period 2018 to 2040) and by 821,309 for prostate cancer (PCa) [1] for the same period.

In Ireland, according to the National Cancer Registry, 1 in 10 women and 1 in 8 men are at risk of BCa and PCa diagnosis by the age of 74, respectively, and about 30% of all invasive cancers [2]. Although the cumulative lifetime risk of diagnosis is high, the cumulative lifetime risk of death by the age of 74 is 1 in 51 for BCa and 1 in 115 for PCa.

There are different drugs currently used to treat BCa and PCa, and new ones being developed. Treatments used in breast cancer include chemotherapy, surgery, radiotherapy, endocrine therapy, and recently targeted therapy and immunotherapy. Treatments used in prostate cancer include endocrine therapy, surgery, chemotherapy, radiotherapy, and Radium Ra 223 dichloride. Bisphosphonates are used in cases when cancer has metastasized to bones. All these treatments are used in combination with each other in order to cure or control the disease. 

In this article, we present the results of a study run at Beacon Hospital. The study’s main aim was to understand and analyze the care pathways (CPs) for BCa and PCa patients and to evaluate the association between different treatment lines, the lifestyle and demographic characteristics of these patients.

## 2. Materials and Methods

### 2.1. Study Design

The study was performed at Beacon Hospital, a private hospital in Dublin, Republic of Ireland. There were 117 patients selected to be contacted to participate in the study. In total, 83 patients agreed to participate and were interviewed, 41 BCa patients and 42 PCa patients. See Appendix A Table A1 for a complete breakdown of all the participants’ involvement thought out the study.

The inclusion criteria were the following: participants ought to be over the age of 18 years; ought to have the capacity to provide informed consent themselves; their current diagnosis ought to be breast cancer or prostate cancer of any cancer stage and care period; they were able to understand and speak English; and they were willing to participate in the study. Exclusion criteria: participants who had been involved in other research projects for the last 8 weeks (2 months) were excluded from the study. This exclusion criterion was included to not burden the patients.

### 2.2. Data Sources

**Figure 1:**
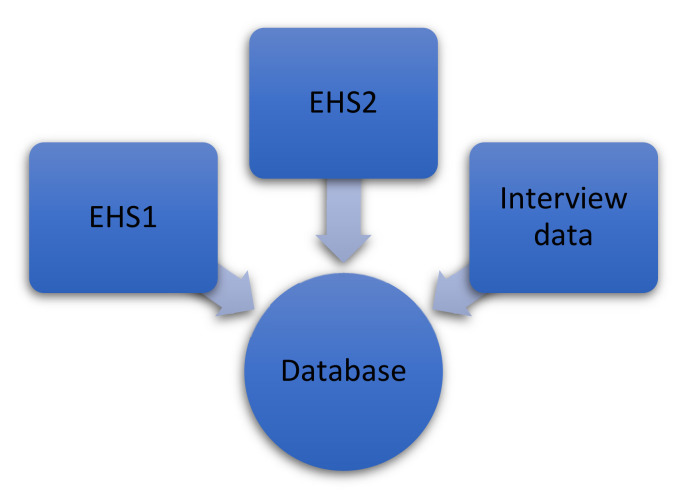
The dataset contains information gathered from two electronic healthcare systems (EHSs) and the qualitative study conducted between January and November 2018.

Demographic, medical, and lifestyle data of the participants were collected through one-on-one interviews with patients performed by the principal investigator (who had no prior knowledge about any of the participants) and electronic health records (EHRs). EHRs were retrieved manually for each patient from both systems used in the hospital. The difference between these systems was that one system was used only in the radiotherapy department (EHS2) and stored only information related to radiotherapy treatment, and the other system was used in the rest of the hospital (EHS1). After we categorized the data retrieved from the interviews and created the EHRs dataset, we combined all the data together, the EHRs and the interview data (Figure 1).

#### 2.2.1. Demographic Data 

Demographic data included the participant’s current age, age at diagnosis, date of birth, province, marital status, education, employment, and religion. The information for the first 3 variables, age, age at diagnosis, and date of birth, was always found in EHRs. The information about the rest was retrieved through interviews. Marital status and religion could be found in EHRs, although most of the time were missing. Participants’ demographic characteristics are presented in Table 1.

#### 2.2.2. Medical Data

Medical data were collected mainly from EHRs in both systems (EHS1 and EHS2). These included hearing, vision, allergies, the diagnosis plan, date of biopsy, biopsy results: type of cancer, grade, stage, progesterone receptor and the score, estrogen receptor and the score, HER2 receptor and the score, tumor size, lymph node involvement, Oncotype score; treatment lines including surgery date, type of surgery, side of surgery; chemotherapy drug, the number of cycles, start and end date of chemotherapy, the status of chemotherapy; radiation site, number of sessions of radiotherapy, the gray unit, start and end date of radiation treatment and status; endocrine therapy drug, dose, start and end date of endocrine therapy, and status; targeted therapy drug, number of cycles, start and end date, and status; immunotherapy drug, number of cycles, start and end date, and status; bisphosphonate drug, number of cycles, start and end date, and status; care phase during the interview, and comorbidities when the data was collected. All the information about the medical aspect was collected consulting different sections in both systems and sometimes even the participant’s patient folder. This was done to get the correct number of chemotherapy cycles and endocrine therapy treatment because it was found that this information was sometimes missing from the EHRs. Participants were asked about their treatment during the interviews, so that we had their views on treatment as well. 

#### 2.2.3. Lifestyle Data

Lifestyle data were collected from the interviews. This included diet, exercise, smoking, alcohol consumption, support system. Such information was sometimes stored indirectly in EHS1 for a limited number of participants, mainly prostate cancer patients. Financial information was retrieved in the form of insurance they used during their care in the hospital. Due to some participants being in their follow-up phase, the insurance information was changed to Self-pay, and the exact insurance coverage during their treatment is not known. However, as the hospital is a private one, all participants had private insurance. Participant’s family history with cancer was collected, and this information was mainly retrieved from interviews. Breast cancer participants were asked about their parity, as nulliparity is one of the risk factors for breast cancer [3].

During the interviews, participants were asked to elaborate specifically on their diet, alcohol intake, smoking, and exercise habits. The Centre for Disease Control categories were used to group the smoking habit [4] into “never smoker,” “former smoker,” “current smoker”. Alcohol consumption was categorized according to the National Institute on Alcohol Abuse and Alcoholism [5] as “never drinker,” “social drinker” (moderate drinker), and “heavy drinker”. Diet and exercise habits were found to be more complex to categorize. Diet was categorized into poor, moderate, and healthy, and it was calculated based on the description of a participant’s typical day. Participants were asked about their diet before and after the cancer diagnosis. Participants who expressed having a diet consisting mainly of ready meals and fast food were considered in the poor diet category. Participants with a mixed diet, a diet that consisted of occasional vegetables and fruits, fish or meat, and once per week of ready meals or fast food would be considered in the moderate diet category. Participants who had a mixed diet with a variety of food consisting mainly of vegetables, fruits, fish, less or no meat would be considered in the healthy diet category. These participants would sometimes grow their own vegetables and were recorded to follow some dietary programs designed for cancer patients after their diagnosis. Participants would describe these dietary programs as eliminating sugary food, animal-based food such as dairy and meat. The exercise habit was grouped into “sedentary”, “low”, “moderate”, and “high” activity. Participants were asked about their daily activity before and after the cancer diagnosis. The sedentary category was described as no activity during the day apart from walking short distances within the house. The low category was described as little activity in the form of walking and/or golfing once a week or gardening. The moderate category was described as going for long walks more than twice per week, running or going to the gym at least once per week, or swimming. The high category was described as doing more than one sport during a weeks’ time. Usually, these participants would be everyday runners, would go to the gym or do water aerobics, long-distance cycle, go hiking, as well as the other activities mentioned in the abovementioned categories.

### 2.3. Statistical Analysis

Descriptive statistics were used to summarize and initially inspect the distributions of the study variables. We used IBM SPSS Statistics version 26 (IBM Corp., Armonk, NY, USA) to analyze the data. The Kruskal–Wallis test was used for numerical variables and Pearson’s chi-square test for categorical variables. Regarding missing observations, SPSS has the option to include or exclude them, and we opted for exclusion. 

### 2.4. Ethics Statement

This research was approved by the Research Ethics Committee at Beacon Hospital, study reference number BEA0084, and the Research Ethics Committee at the University of Deusto, study reference number ETK-08/17-18.

## 3. Results

### 3.1. Breast Cancer Study

The total number of variables in the breast cancer dataset was *p* = 163. The total number of distinctive participants was 41, 4 of whom had a cancer recurrence, and one was diagnosed with two different types of breast cancer, invasive ductal carcinoma and invasive lobular carcinoma. Some variables, *p* = 38, were removed from the analysis because they presented with more than 90% of the data missing. The plan for the staging variable represents the triple assessment care pathway, which includes a mammogram, an ultrasound, and a biopsy examination. Some of the patients had other examinations performed during the diagnosis stage, such as MRI and CT scans, one of the reasons for this being breast density. Research has shown that for patients with dense breasts, a supplemental MRI is needed [6]. The data indicate that 8/46 patients underwent more examinations than stated in the triple assessment care pathway. The care pathway for non-metastatic breast cancer followed at Beacon Hospital can be found in Table A2.

Before the treatment started, 36/46 went through further examinations, with most common being CT TAP (27) and MRI (18), followed by the Nuclear Medicine bone scan (11). 

The average waiting time to start the treatment was 20.65 days; 78.3% (n = 36) started their treatment within 31 days (one month). The other 10 participants began their treatment within 50 days after their diagnosis. Of the 10 participants, 6 were diagnosed with metastasized breast cancer. Four out of six had cancer metastasized to bones, one in the liver, and the other patient was initially diagnosed with cancer of unknown primary metastasized to the breast. The prolonged start of treatment was due to further examinations to see the extent of the metastasis. However, there were cases where the delay was requested by patients. 

In this analysis, we wanted to see how different lines of treatment were affected by the input variables we collected. For this reason, we ran the following tests to see which of the input variables played a role in whether a BCa patient had chemotherapy, radiotherapy, surgery, targeted therapy, immunotherapy, and bisphosphonate; see Table 2. 

#### 3.1.1. Chemotherapy

The most common chemotherapy combinations were doxorubicin, cyclophosphamide, and docetaxel or paclitaxel (AC + T), docetaxel or paclitaxel, carboplatin and trastuzumab (TCH), and cyclophosphamide, methotrexate, and fluorouracil (CMF). The test showed that there was a statistically significant difference in the patient’s age at diagnosis (95% Confidence Interval (CI) 51.39–58.92, *p* = 0.047) and hearing (95% CI −0.17–0.42, *p* = 0.029) between the group that was treated with chemotherapy and the other one that was not. The age at diagnosis showed that patients aged 65 (mean) were less predisposed to receive chemotherapy than those aged 55 (mean). 

#### 3.1.2. Targeted Therapy

The test showed that there was a statistically significant difference in the patient’s HER2 score (95% CI 1.99–2.92, *p* < 0.01), chemotherapy cycles (95% CI 4.22–8.38, *p* = 0.012), and the smoking habits (95% CI 0.20–0.50, *p* = 0.038). Targeted therapy is especially administered when the patient is diagnosed with HER2 positive breast cancer, and our analysis confirmed the same. It was observed that patients who did not receive targeted therapy had never smoked, and patients who received targeted therapy tended to have more chemotherapy cycles.

#### 3.1.3. Surgery 

The test showed that there was a statistically significant difference in patient’s diet (95% CI 1.46–1.85, *p* = 0.016), exercise (95% CI 1.20–1.96, *p* = 0.029), and chemotherapy cycles (chemotherapy cycles 1: 95% CI 5.06–6.33, *p* = 0.007 and chemotherapy cycles 2: 95% CI 5.06–6.33, *p* = 0.005). Patients who had a moderate to a healthy diet and moderate to high active lifestyle were shown to have been more likely to have undergone surgeries compared to patients who had self-reported being less active and had a poorer diet.

#### 3.1.4. Endocrine Therapy

In this cohort study, the majority of patients started their endocrine therapy treatment after they had successfully completed other treatments such as surgery, chemotherapy and/or radiotherapy treatments. Endocrine therapy is usually prescribed to be taken for a period of 3 to 5 years. The analysis showed that there was a statistically significant difference in patient’s years with cancer (95% CI 1.63–4.37, *p* = 0.016). This treatment line is usually prescribed to patients who have been diagnosed with hormone-positive receptors: the progesterone receptor score (95% CI 3.08–6.447, *p* = 0.016) and estrogen receptor score (95% CI 5.19–7.22, *p* < 0.001). A relation was observed between chemotherapy cycles and endocrine therapy. The more chemotherapy cycles a patient had, the more likely it was that the patient would continue with endocrine therapy (95% CI 4.77–13.59, *p* = 0.044).

#### 3.1.5. Radiotherapy

Radiotherapy is another common treatment for breast cancer patients, alongside chemotherapy and surgery. Patients with a body mass index above normal (BMI > 25) were observed to have a greater number of radiotherapy treatments compared to patients with a smaller BMI (BMI: 95% CI 25.63–30.41, *p* = 0.007). Patients with no radiotherapy treatment were observed to have more chemotherapy cycles (95% CI 3.85–16.65, *p* = 0.049).

#### 3.1.6. Bisphosphonate

Bisphosphonate is a medication used for treating bone diseases. In the case of breast cancer, it is used when the disease has metastasized to the bones. The cancer stage is one of the indicators as to whether the disease has metastasized or not, starting from 0 to 4, 0 being non-invasive BCa. The test showed that there was a statistically significant difference in the patient’s cancer metastasis (stage: 95% CI 4.00–4.00, *p* = 0.022). Patients that underwent bisphosphonate treatments underwent more chemotherapy cycles (more than eight cycles of chemotherapy) compared to patients that did not have bisphosphonate treatment (these patients usually had less than eight chemotherapy cycles) (chemo_cycles1: 95% CI 5.19–6.60, *p* = 0.012; chemo_cycles2: 95% CI 4.99–7.51, *p* = 0.028).

The most common first line of treatment was chemotherapy (n = 19), followed by surgery (n = 18). The other treatments were less common: endocrine therapy (n = 4) and radiotherapy (n = 3). The most common second lines of treatment were chemotherapy (n = 18) and targeted therapy (n = 14). The most common third lines of treatment were radiotherapy (n = 11), target therapy (n = 9), and surgery (n = 8). The most common fourth lines of treatment were endocrine therapy (n = 11) and radiotherapy (n = 9). Metastasized BCa patients received more than five lines of treatments. In total, 32 participants had surgery, chemotherapy (n = 38), targeted therapy (n = 25), radiotherapy (n = 28), endocrine therapy (n = 26), bisphosphonate (n = 11), and immunotherapy (n = 1).

### 3.2. Prostate Cancer Study

The total number of variables in the prostate cancer dataset was *p* = 77, and the number of patients was n = 42. Twenty-nine participants were diagnosed with metastatic PCa, 22 of them with bone metastasis. Cancer had spread to other organs such as the bladder, pelvis, liver, mesorectum, and lumbar spine. We did not have information on the metastasized organ for five participants.

There was no care pathway for PCa. PCa patients were diagnosed together with other urologic diseases in the Urology department. The plan for staging was very different from one participant to another. We did not have information for six participants; however, 24 participants had undergone at least three examinations (n = 36 at least one, and n = 32 at least two examinations). The three most common examinations were biopsy (27/42), MRI (21/42), and nuclear bone scan (15/42). Before the treatment started, 19/42 went through further examinations, the most common being CT (10) and MRI (6). 

The average waiting time to start the treatment was 19.5 days; 78.6% (n = 33) started their treatment within 31 days (one month). The other nine participants began their treatment within 4 months after their diagnosis. Of the nine participants, six were diagnosed with metastasized PCa. Four out of six had cancer metastasized to bones.

Specific results are described below, while all the results are presented in Table 3.

#### 3.2.1. Endocrine Therapy

Endocrine therapy was the most common treatment for PCa patients in the hospital. Every patient had endocrine therapy. For this reason, no significant difference was observed to play any role in this treatment. 

#### 3.2.2. Chemotherapy

Docetaxel was the only chemotherapy drug used to treat prostate cancer patients. It was observed that taller (height_cm: 95% CI 176.70–183.85, *p* = 0.005), heavier (weight_kg: 95% CI 85.80–99.57, *p* < 0.001) patients were more likely to have chemotherapy compared to the ones who were shorter, lighter, and with BMI less than 25. 

#### 3.2.3. Radiotherapy

Radiotherapy is a widespread treatment for different types of PCa, and especially for prostate cancer that has metastasized. In such cases, patients who had some discomforts would undergo one to two sessions to manage the symptoms. Our results showed that patients who had been diagnosed with PCa for more than 2 years were more likely to undergo radiotherapy treatments compared to those who had been diagnosed for less than 2 years (95% CI 3.19–6.49, *p* = 0.003). There were some outliers, but these patients were in their follow-up phase, meaning they had completed their treatment. As in other treatments, height and weight variables showed to be significant. Shorter patients were more likely to undergo radiotherapy treatments, compared to taller ones (95% CI 170.42–176.53, *p* = 0.01). Heavier patients were less likely to undergo radiotherapy compared to those who weighed less than 83 kg (95% CI 73.69–84.92, *p* = 0.007). 

#### 3.2.4. Bisphosphonate

This drug is usually administered in patients who are diagnosed with metastatic cancer. In our study, the analysis showed that patients with an initial prostate-specific antigen level (PSA level) above 25 were more likely to be treated with this drug (initial_psa: 95% CI 45.51–96.14, *p* = 0.002).

#### 3.2.5. Radium Ra 223 Dichloride 

Radium Ra 223 dichloride is a drug used to treat prostate cancer that has spread to the bone and is causing symptoms but has not spread to other organs. It is used in patients whose cancer is castration-resistant (cancer that keeps growing even when the amount of testosterone in the body is reduced to very low levels). Our analysis showed that patients with poor diet tended to be the ones who had Radium Ra 223 dichloride treatments (95% CI −0.51–1.71, *p* = 0.057).

#### 3.2.6. Surgery

This treatment was the less preferred treatment. Many opted for less invasive treatments such as endocrine therapy and chemotherapy. Although the number of participants in the study was small (n = 42), the analysis showed that participants who had been diagnosed with prostate cancer within 5 years (95% CI 2.72–10.95, *p* = 0.015) and had a sedentary or low active lifestyle (95% CI −0.21–0.88, *p* = 0.02) were more predisposed to undergoing surgery. 

The most common first line of treatment for PCa participants was endocrine therapy (n = 33), followed by radiotherapy (n = 6). The most common second-line treatments were endocrine therapy (n = 22) and radiotherapy (n = 7). The most common third lines of treatment were endocrine therapy (n = 14) and bisphosphonate (n = 10). The most common fourth lines of treatment were endocrine therapy (n = 12) and bisphosphonate (n = 10). Patients with five lines of treatment and more used other treatments such as chemotherapy, radiotherapy, and Radium Ra 223 dichloride. In total, seven participants had surgery, chemotherapy (n = 17), radiotherapy (n = 24), endocrine therapy (n = 41), bisphosphonate (n = 33), and Radium Ra 223 dichloride (n = 8).

### 3.3. Breast and Prostate Analysis

Both datasets were combined to see if there was any significant difference between groups. We excluded the treatments that were used in only one of the groups, i.e., targeted therapy and Radium Ra 223 dichloride. 

#### 3.3.1. Chemotherapy

As was observed in the groups independently, the age of diagnosis played a role in whether a patient had chemotherapy or not. Patients above 70 years of age did not have chemotherapy, whereas patients younger than 70 (median age 59) had chemotherapy (95% CI 55.70–62.18, *p* < 0.001). If the patients were diagnosed within the last 2 years, they had chemotherapy (95% CI 1.25–2.31, *p* < 0.001). Patients with higher BMI (BMI > 25) had chemotherapy, compared to those with a BMI < 25 (95% CI 26.02–28.96, *p* = 0.046).

#### 3.3.2. Radiotherapy

No significant differences were observed apart from a tendency of participants with an average height of 170 or more, who were less likely to receive radiotherapy compared to those with a height of less than 170 cm (mean 168.28) (95% CI 165.88–170.67, *p* = 0.076).

#### 3.3.3. Endocrine Therapy

Although no significance was observed in the prostate cancer group, the combined analysis showed that the older the patients when they were diagnosed, the more likely they were to have endocrine therapy treatment (95% CI 61.30–67.19, *p* = 0.05) (this was observed even when the current age of the participants was used instead (95% CI 64.28–70.48, *p* = 0.026)). The analysis showed that taller (height 170 cm (average) and above) and heavier (weight 81 kg (average) and above) patients were more likely to have endocrine therapy compared to shorter and less heavy patients (height_cm: 95% CI 169.42–174.10, *p* = 0.003; weight_kg: 95% CI 75.14–83.71, *p* = 0.044). 

#### 3.3.4. Surgery

The test showed that there was a statistically significant difference in the patient’s height. Shorter patients (height 170 and below) were more likely to undergo surgery compared to taller patients (95% CI 164.15–169.38, *p* = 0.005). 

#### 3.3.5. Bisphosphonate

Older patients (average age 66) were more likely to have bisphosphonate treatment, compared to younger patients (95% CI 62.88–69.63, *p* = 0.006). Similar to other treatments, the test showed that there was a statistically significant difference in patient’s height and weight, with taller and heavier patients having bisphosphonate treatment compared to shorter and less heavy patients (height_cm: 95% CI 164.33–169.73, *p* = 0.003; weight_kg: 95% CI 68.50–78.65, *p* = 0.027).

## 4. Discussion

In this study, we wanted to analyze different treatment lines that BCa and PCa patients underwent while being treated at Beacon Hospital. We analyzed the following treatment lines for BCa: chemotherapy, radiotherapy, endocrine therapy, surgery, targeted therapy, and bisphosphonate. We analyzed the following treatment line for PCa: chemotherapy, endocrine therapy, surgery, radiotherapy, bisphosphonate, and Radium Ra 223 dichloride. Chemotherapy, radiotherapy, surgery, endocrine therapy, and bisphosphonate were analyzed for both groups. Immunotherapy treatment was used by only one BCa participant, and Ipatasertib, an experimental drug, was used by only one PCa participant. For this reason, they were excluded from the analysis. 

Our analysis for BCa participants showed the relevance of several lifestyle factors, consistent with previous studies [7,8,9,10,11,12]. Additionally, the number of chemotherapy cycles indicated whether a patient underwent other types of treatments such as targeted therapy, surgery, endocrine therapy, radiotherapy, or bisphosphonate. It must be noted that chemotherapy is one of the main treatments for breast cancer. It is used in both non-metastatic and metastatic diseases, compared to surgery, which is still under review on metastatic disease [13,14].

On the other hand, we saw relations that have been proven by the scientific community, such as the relation between the HER2 score and targeted therapy. Targeted therapy drugs such as trastuzumab and pertuzumab are specifically created to target human epidermal growth factor receptor 2 (HER2 receptor) [15].

Endocrine therapy drugs, taken in the form of tablets, are treatments specifically targeting progesterone and estrogen receptors. These drugs are prescribed to be taken for an extended period of time, 3 to 5 years or even longer periods [16,17]; hence, there will be a strong relationship between the number of years a patient is diagnosed with cancer and endocrine therapy treatment. According to the data collected from the study for non-metastatic breast cancers, endocrine therapy is the last treatment prescribed to patients. Thus, a newly diagnosed patient will have to go through other treatments first, which usually takes up to 1 year, before starting with endocrine therapy. 

**Figure 2:**
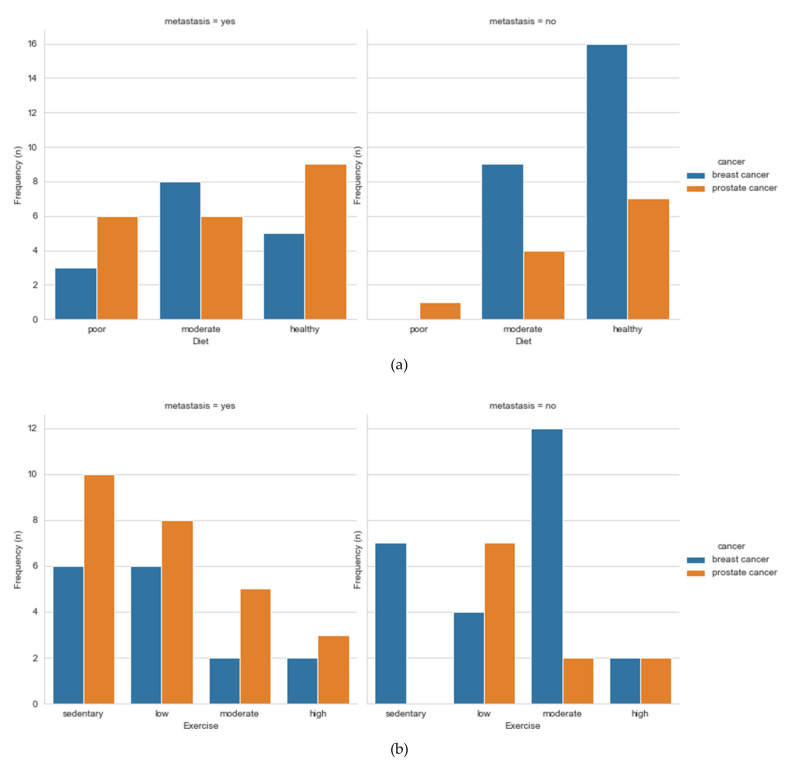

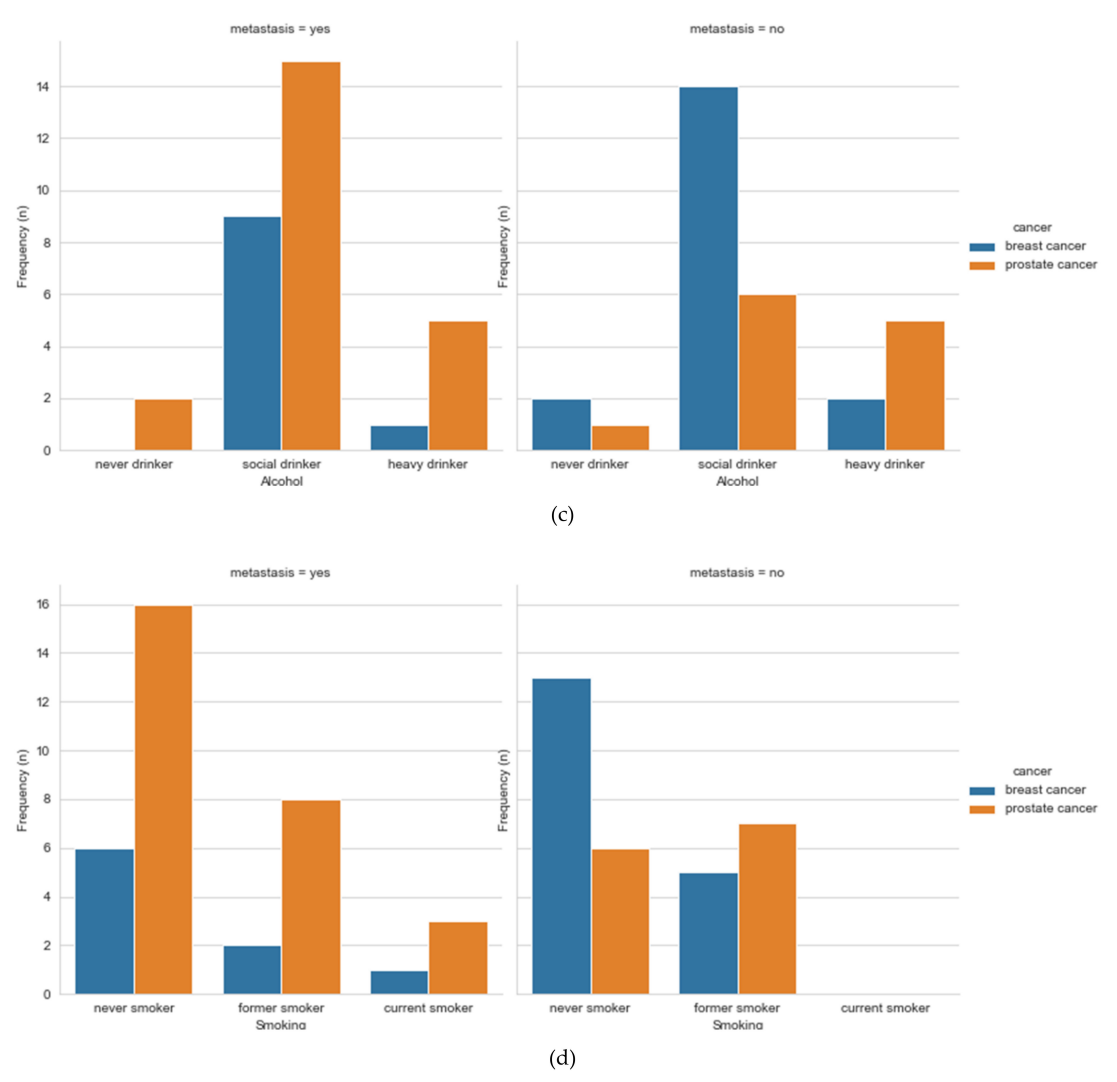
Population graphs for BCa and PCa diet (**a**), exercise (**b**), alcohol (**c**), and smoking (**d**) for metastatic and non-metastatic disease.

**Figure 3:**
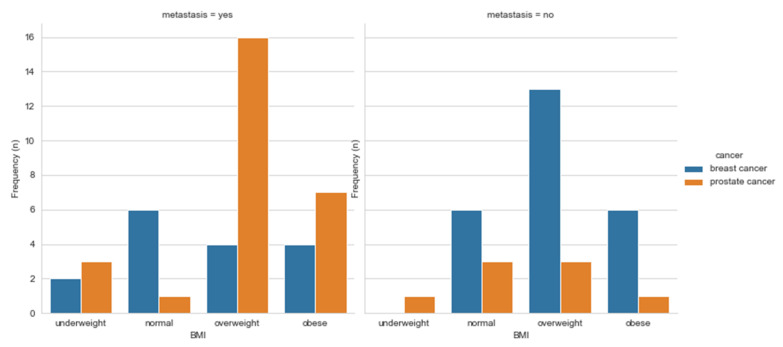
Population graphs for BCa and PCa BMI for metastatic and non-metastatic disease.

Our data showed some relations between lifestyle variables such as diet (Figure 2a), exercise (Figure 2b), alcohol (Figure 2c), and smoking (Figure 2d). A healthy diet and an active life usually mean a normal BMI. In our data, we observed that patients with higher BMI (BMI > 25), especially PCa patients, are diagnosed with metastatic cancer, Figure 3. Studies have shown that, indeed, obese men tend to be diagnosed with advanced PCa, compared to those with normal BMI [18]. Similar was observed with weight changes. Bigger weight gains were associated with a higher risk of aggressive PCa [19]. Recent systematic reviews have shown no clear association between BMI and PCa, but a strong inverse association between BMI and PSA [20]. The same was observed for sedentary behavior; however, sedentary behaviors are considered as modifiable behavior risk factors through the mechanism involving obesity for aggressive PCa [21]. Seventeen out of 41 BCa participants were diagnosed with metastatic disease, and only 5 of them had undergone surgery. To the best of our knowledge, no study has been published to see whether healthier and fitter patients are more likely to undergo surgeries compared to less healthy and active patients. More studies should be done in this aspect to check whether these results are true in other situations, i.e., bigger study samples, other countries, etc. 

**Figure 4:**
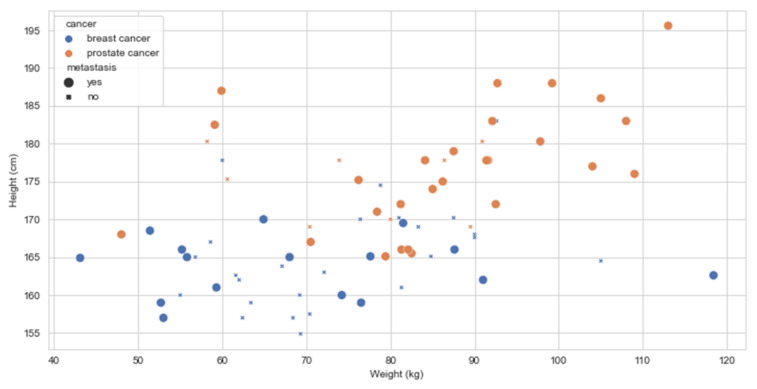
Population graphs for BCa and PCa BMI for metastatic and non-metastatic disease.

**Figure 5:**
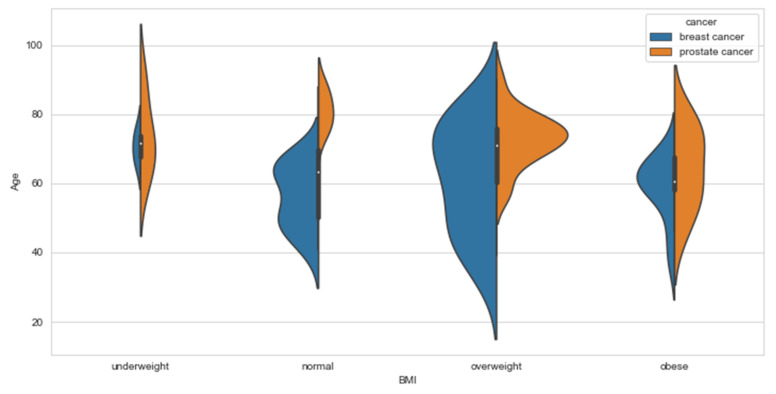
Population graphs for BCa and PCa BMI for metastatic and non-metastatic disease.

In the analysis of the PCa data, height and weight are observed to play different roles when it comes to having chemotherapy and radiotherapy. The relation between height, or tallness, and prostate cancer has been studied for many years. Tall height is associated with an increased risk of high-grade PCa and in PCa mortality [22,23,24,25]. Our analysis showed height and weight play some role if a patient has a specific type of treatment. Figure 4 shows the height and weight of all participants in the study, indicating that taller and heavier patients are diagnosed with metastatic disease. In Figure 5, we compare the distribution of age across all BMI groups between BCa and PCa.

We have to acknowledge several limitations in this study. The number of participants was small, and represented people with good economic status (all participants had private insurance). Most participants, n = 77, were from the capital region, which does not represent the entire country of the Republic of Ireland. Few of the participants were diagnosed and/or had started their treatment in another hospital before transferring their care to Beacon Hospital. However, these patients were treated by the same doctors, and most of these patients’ previous treatments were recorded as well. The start and finish dates of chemotherapy, radiotherapy, endocrine therapy, and bisphosphonate are approximations, not the exact dates. We did not have information for every patient about the status of their treatment, finished or discontinued. The dataset is not balanced. It does not have an equal number of participants between different age groups, cancer metastasis, or between different care phases (treatment, follow-up, palliative).

## 5. Conclusions

We believe studying different types of treatment a patient has during his/her cancer journey is very important. The size of our study was small, and the results may differ if we conduct a similar study in another country, but it is worth studying what treatments are being used and what role different factors play in these treatments.

## Figures and Tables

**Table 1:** Participant characteristics.

Characteristics	Breast Cancer	Prostate Cancer
Sex		
Male	0 (0%)	42 (100%)
Female	41 (100%)	0 (0%)
Age (years)		
Median (range)	61 (33–83)	74 (46–90)
Median on diagnosis (range)	58 (33–81)	66 (38–86)
Education		
No education	0 (0%)	2 (4.8%)
Primary school	0 (0%)	5 (11.9%)
Secondary school	12 (29.3%)	4 (9.5%)
Professional certificate	1 (2.4%)	6 (14.3%)
Bachelor’s degree	20 (48.8%)	9 (21.4%)
Master’s degree	8 (19.5%)	1 (2.4%)
PhD	0 (0%)	4 (9.5%)
No information	0 (0%)	11 (26.2%)
Work		
Full time employment	18 (43.9%)	6 (14.3%)
Part time employment	1 (2.4%)	2 (4.8%)
Unemployed	2 (4.9%)	2 (4.8%)
Retired	19 (46.3%)	25 (59.5%)
No information	1 (2.4%)	7 (16.7%)
Marital status		
Single	5 (12.2%)	3 (7.1%)
Married	24 (58.5%)	34 (81.0%)
Partnership	2 (4.9%)	0 (0%)
Widowed	6 (14.6%)	5 (11.9%)
Divorced	1 (2.4%)	0 (0%)
Unmarried	3 (7.3%)	0 (0%)
Provinces		
Connacht	0 (0%)	2 (4.8%)
Leinster	40 (97.6%)	37 (88.1%)
Munster	1 (2.4%)	1 (2.4%)
Ulster	0 (0%)	2 (4.8%)
Religion		
Not religious	7 (17.1%)	2 (4.8%)
Religious	25 (61.0%)	30 (71.4%)
Not disclosed	9 (22.0%)	10 (23.8%)
Insurance		
Private insurance	24 (58.5%)	37 (88.1%)
Self-pay	17 (41.5%)	5 (11.9%)

**Table 2:** Variables used in the analysis of different treatment lines for breast cancer.

	Chemotherapy (38/46)	Radiotherapy (28/46)	Targeted Therapy (25/46)	Endocrine Therapy (26/46)	Surgery (32/46)	BISPHOSPHONATE (11/46)	Immunotherapy (1/46)
Age group	0.079	0.506	0.837	0.527	0.638	0.708	0.876
Hearing	0.029	0.423	0.359	0.380	0.508	0.575	0.881
Vision	0.363	0.734	0.596	0.596	0.075	0.267	0.371
Allergies	0.496	0.801	0.773	0.191	0.497	0.435	0.458
BMI groups	0.509	0.044	0.972	0.474	0.411	0.531	0.282
Tumor stage group	0.830	0.834	0.498	0.588	0.169	0.146	.^a^
Cancer grade	0.200	0.528	0.169	0.212	0.134	0.058	.^a^
Cancer stage	0.628	0.404	0.272	0.279	0.058	0.022	0.414
Diet	0.522	0.919	0.221	0.132	0.016	0.051	0.067
Alcohol	0.131	0.276	0.374	0.096	0.654	0.147	0.026
Smoking	0.244	0.170	0.038	0.918	0.095	0.710	0.182
Exercise	0.639	0.934	0.704	0.635	0.029	0.087	0.208
Age at data collection	0.039	0.752	0.612	0.723	0.867	0.887	0.706
Age at diagnosis	0.047	0.735	0.635	0.807	0.877	0.787	0.851
Years diagnosed with cancer	0.333	0.394	0.769	0.016	0.076	0.310	0.316
Height (cm)	0.632	0.089	0.154	0.649	0.693	0.511	0.546
Weight (kg)	0.739	0.042	0.675	0.471	0.346	0.797	0.243
BMI	0.783	0.007	0.974	0.492	0.527	0.787	0.163
Tumor size (mm)	0.706	0.784	0.503	0.800	0.331	0.174	.^a^
Progesterone receptor score	0.438	0.862	0.886	0.016	0.365	0.279	0.295
Estrogen receptor score	0.266	0.830	0.747	0.000	0.456	0.740	0.197
HER2 score	0.690	0.708	0.000	0.715	0.312	0.229	0.154
Days between diagnosis and starting of treatment	0.542	1.000	0.860	0.002	0.189	0.374	0.090
Chemotherapy 1 cycles	0.953	0.049	0.012	0.044	0.007	0.012	0.114
Chemotherapy 2 cycles	0.843	0.171	0.103	0.340	0.005	0.028	0.048
Chemotherapy 3 cycles	.^a^	0.881	0.319	0.811	0.319	0.319	.^a^
Targeted therapy cycles	0.662	0.951	.^a^	0.653	0.343	0.847	.^a^
Bisphosphonate cycles	0.813	0.170	0.260	0.184	0.238	0.045	.^a^
Tumor size post neoadjuvant chemotherapy (mm)	0.353	0.353	0.715	0.172	0.353	0.353	.^a^
Radiotherapy sessions first site	0.139	0.812	0.575	0.330	0.355	0.084	0.317
Radiotherapy site one Grey units	0.622	0.579	0.227	0.471	0.234	0.084	0.317
Radiotherapy sessions 2nd site	0.376	0.274	0.741	0.804	1.000	0.510	0.302
Radiotherapy site two Grey units	0.189	0.829	0.683	0.288	0.392	0.780	0.307
Oncotype score	.^a^	0.737	.^a^	0.764	0.380	.^a^	.^a^

^a.^ There is only one group with valid data. Asymptotic significances (2-sided test) are displayed. The significance level is 0.050.

**Table 3:** Variables used in the analysis for different treatment lines for prostate cancer.

	Chemotherapy (17/42)	Radiotherapy (24/42)	Surgery (7/42)	Bisphosphonate (33/42)	Xofigo (8/42)
Age	0.002	0.088	0.332	0.198	0.577
Age at diagnosis	0.053	0.380	0.855	0.165	0.543
Years with cancer	0.000	0.003	0.015	0.501	0.633
Height (cm)	0.005	0.010	0.427	0.482	0.878
Weight (kg)	0.000	0.007	0.559	0.310	0.444
BMI	0.095	0.371	0.725	0.274	0.457
Initial PSA	0.840	0.097	0.820	0.002	0.185
Diagnosis to treatment days	0.040	0.523	0.000	0.592	0.397
Age group	0.067	0.923	1.000	0.281	0.741
BMI groups	0.017	0.362	0.430	0.482	0.455
Gleason score	0.490	0.215	0.347	0.495	0.207
Diet	0.810	0.281	0.434	0.483	0.057
Drinking	0.222	0.920	0.976	0.613	0.926
Smoking	0.693	0.901	0.458	0.350	0.653
Exercise	0.562	0.811	0.020	0.679	0.141

Asymptotic significances (2-sided test) are displayed. The significance level is 0.050.

## Data Availability

The data presented in this study are available on request from the corresponding author.

## Appendix A

**Table A1:** Patients selected and contacted for the study.

Patients	No. of Patients
Total number of patients selected to participate in the study	117
Patients contacted	109
Patients participated	83
Breast cancer patients	41
Prostate cancer patients	42
Patients who did not want to participate	18
Patients who withdrew	1
Patients who forgot about the interview appointment	1
Patients who rescheduled the interview beyond the study period	1
Patients who did not feel well to participate	4
Patients who did not participate because of the length of the interview	4
Patients who did not participate and did not give any explanation	7
Patients who were told about the study but were not interviewed because the number of participants was reached	8
Patients who were selected but not contacted	8

**Table A2:** Care pathway for non-metastatic breast cancer patients.

**Pre diagnosis** Hospital referral
**Diagnosis** Breast clinic appointment Triple assessment (TA): physical examination, mammogram, ultrasound, biopsy (same day) MRI a few days after TA if dense breast or other reasons Results Biopsy results and scans Verbal and written information about cancer A preliminary care plan, awaiting the receptor status results, described to the patient during clinic visit defining the first line of treatment Results for receptor status: progesterone, estrogen and HER2 status defining the course of treatment Contact information at the hospital Information about the importance of exercise Information about fertility for young adult patients
**Treatment** 6 weeks rest between each treatment Information about the importance of exercise Information about fertility for young adult patients Chemotherapy Information about chemotherapy Chemotherapy side effects and how to mitigate them Surgery Information about the surgery Length of stay at the hospital after surgery Physiotherapy sessions: to recover from surgery and to prepare for radiotherapy Radiotherapy: Information about radiotherapy and its side effects Radiotherapy measurements Contact information at the radiotherapy department Endocrine therapy: Information about the endocrine treatment and its side effects
**Follow-up** Six-month follow-up after the surgery Six-month follow-ups with the medical oncologist Yearly mammogram Yearly appointments with the gynecologist
